# A study on deep learning model based on global–local structure for crowd flow prediction

**DOI:** 10.1038/s41598-024-63310-6

**Published:** 2024-06-01

**Authors:** HeounMo Go, SangHyun Park

**Affiliations:** Department of Computer Science, Yonsei University, Yonsei-ro 50, Seodaemun-gu, Seoul, 03722 Republic of Korea

**Keywords:** Crowd flow prediction, Deep learning, Spatio-temporal data mining, Computer science, Information technology

## Abstract

Crowd flow prediction has been studied for a variety of purposes, ranging from the private sector such as location selection of stores according to the characteristics of commercial districts and customer-tailored marketing to the public sector for social infrastructure design such as transportation networks. Its importance is even greater in light of the spread of contagious diseases such as COVID-19. In many cases, crowd flow can be divided into subgroups by common characteristics such as gender, age, location type, etc. If we use such hierarchical structure of the data effectively, we can improve prediction accuracy of crowd flow for subgroups. But the existing prediction models do not consider such hierarchical structure of the data. In this study, we propose a deep learning model based on global–local structure of the crowd flow data, which utilizes the overall(global) and subdivided by the types of sites(local) crowd flow data simultaneously to predict the crowd flow of each subgroup. The experiment result shows that the proposed model improves the prediction accuracy of each sub-divided subgroup by 5.2% (Table 5 Cat #9)—45.95% (Table 11 Cat #5), depending on the data set. This result comes from the comparison with the related works under the same condition that use target category data to predict each subgroup. In addition, when we refine the global data composition by considering the correlation between subgroups and excluding low correlated subgroups, the prediction accuracy is further improved by 5.6–48.65%.

## Introduction

A significant amount of spatial-temporal data is being generated across many areas and the applications of such data are getting more important from target marketing to urban planning and public safety. In this paper, we focus on crowd flow density prediction, one of the spatial–temporal data prediction. Last year, in South Korea, there was a massive crowd crush in Seoul’s Itaewon neighborhood and left many people dead and injured. If there was a public safety system based on crowd flow prediction, the tragedy could be avoided. As similar tragedies occur every year, the importance of crowd flow prediction is evident. As location-based services using smartphones become popular, research on crowd flow prediction using this data has also been actively conducted. In the private sector, it has been used for location selection of stores and customer-tailored marketing. In the public sector, it has been used for social infrastructure construction such as transportation networks. In public health area, with the recent spread of Covid-19, the crowd flow prediction is also gaining attention to prevent the spread of infectious diseases.

In many cases, the crowd flow data can be divided into subgroups. For example, the population can be divided by age and gender, and data from location based social network services can be also divided into subcategories by the type of visited spots such as food and shopping. In other words, The data exhibits hierarchical structure. To refine the effectiveness of crowd flow predictions, we need to account not only for the entire population but also for each sub-category. For example, for personalized marketing that is tailored to a specific demographic, it would be more effective to predict the movement of that demographic rather than the entire population. We propose a model structure that leverages the hierarchical structural characteristics of the data to maximize its utilization. To the best of our knowledge, our study is the first to concentrate on the hierarchical structure of crowd flow data.

## Related work

There are some previous works on predicting an individual’s movement based on their location history^1,2^. These works focus on an individual, rather than for overall crowd-flow. Due to the growing popularity of deep learning, it has also been applied to crowd flow prediction. Deep learning-based models, such as Deep-ST^3^, which adapts a convolutional neural network^4^ to model the spatial relations, has been proposed. In addition, ST-ResNet^5^ has been suggested by using a residual framework^6^ instead of the general convolution operation. DeepSTN+^7^, which considers the long-range spatial dependency more precisely, has been proposed.

On the other hand, RNN^8^ based models have been suggested because crowd flow data have time-series nature. Models such as ConvLSTM^9^, hybrid approach^10^, STRCN^11^ have been suggested with combining convolution and LSTM. In addition, Periodic-CRN^12^, which combines the pyramidal ConvGRU model with periodic representations to explicitly model the periodic nature of crowd flows, has been designed.

In addition, models specialized for traffic flow prediction in large cities, such as^13,14^, have been proposed. Particularly, novel methods of integrating inherent patterns in traffic flows, such as fixed spatial distributions and topological correlations^15^, and utilizing the GAN technique to predict traffic patterns with incomplete surveillance information^16^, have been proposed.

In the case of fine-grained data sets for metropolitan areas, a CNN-based model for enhancing computational efficiency, such as^17^, has been proposed. It converts the grid-based structure into a region-based one and models the flow between regions, and it shows promising prediction accuracy and computational performance. DeepCrowd^18^ has been proposed, which adapts pyramid convLSTM and advanced attention mechanisms and is applied to fine-grained data sets. GridFormer^19^, which utilizes a periodically shifted sampling method and an attention mechanism to manage period disturbances, along with a pyramid 3D Swin transformers to capture long-range dependencies in a hierarchical fashion, has been proposed.

Additionally, research has been conducted on spatiotemporal multi-factor analysis, suggesting a new model architecture that learns features at all levels and captures spatiotemporal correlations at all-time stages using a modified densely connected convolutional network^20^

However, the existing models do not take into account the hierarchical structure of data described above. For example, when predicting one of the subcategories of data, existing models typically use only the target category data, without fully utilizing all the other categories of data. On the other hand, our novel model utilizes both the aggregated data (global) and the segmented data (local) according to the types of POI, consequently delivering improved performance compared to the existing models. Here, POI refers to Point of Interest, a location that a person visits. In addition, we propose a method for configuring global data that takes into account not only all categories but also the correlation between categories. This method of utilizing the hierarchical structure of global-local data has been previously used for high-dimensional multivariate time series data prediction^21^, but this is the first application of this approach to crowd flow prediction.

## Methods

### Problem formulation

To represent the regions of the city, we divide a city into an H $\times$ W grids by the longitude and latitude, where all grids have the same size and each grid indicates a region. We define crowd flow density of a category for the region (h, w) at the ith time interval as follows:

$$ \begin{aligned} {x_{i}}^{h, w, c} = \mid \{ { j \ge 1 \mid g_j\in (h,w) \;  \&  \; c = category} \} \mid \end{aligned}$$

Here, $g_j$ is the geospatial coordinate; $g_j \in (h, w)$ means the point $g_j$ lies within grid (h, w), and vice versa; $\mid . \mid$ represents the cardinality of a set. c is the category of the point $g_j$ belongs to. The target of crowd flow density prediction is to predict $X_n$, given the historical observations $\{ X_i \mid i = 1, 2,\ldots , n-1 \}$

### Model

In this study, we adopt DeepSTN+^7^ as the base model. This is because it is a CNN-based model that can efficiently identify patterns on the grid structure and is among the most recent models for crowd flow prediction. However, the core idea of this study, which is the global-local structure approach, can be adapted to other models with some modifications.

**Figure 1:**
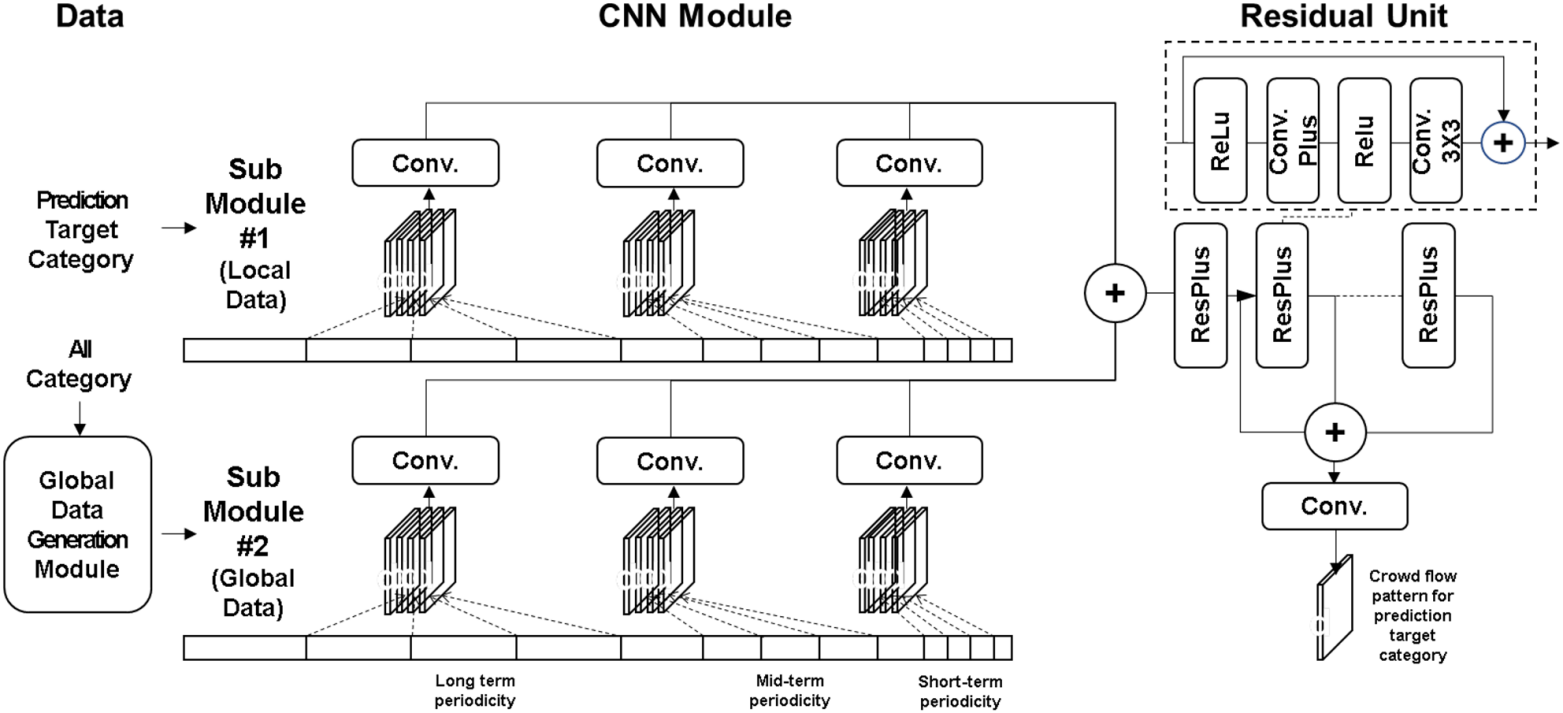
Model architecture.

Figure 1 shows the deep learning model proposed in this study based on global-local structure by leveraging the base model. First, input data is divided into local data, which is the target category data to be predicted, and global data, which includes all or some other categories. The CNN module is split into sub-module 1 for modeling local data and sub-module 2 for modeling global data. Local data is the input of CNN sub-module 1, and global data is the input of the global data generation module. The global data generation module is responsible for consisting global data by either including all categories as they are or removing some low correlated categories from the global data through calculating the correlation between the target category and each other category. Our assumption is that the lower a category is correlated with target category, the more excluding the category from global data improves the prediction performance. More specifically, when considering the correlation between categories, we select the top M categories with the highest correlation to the target category, and any categories with a correlation higher than N are also included. We call N correlation threshold. In this experiment, M and N are selected empirically based on the experimental results. In the case of Foursquare, M = 5 and N = 0.31 are selected because correlations vary significantly between categories, and in Gowalla, M = 8 and N = 1 are chosen as the correlations between categories are relatively high and the values are similar. We propose that, in instances where there are significant differences in the correlations between categories, it is advisable to opt for lower values of M and N. This is primarily because it is generally more beneficial to exclude categories with low correlations. Conversely, when the correlations between categories are high, it is preferable to choose higher values of M and N, as it is desirable to include as many highly correlated categories as possible. In this study, we use pearson correlation.

The subsequent CNN module is split into sub-module 1 for modeling local data and sub-module 2 for modeling global data. Each sub-module is composed of three CNN modules for modeling short-term periodicity, medium-term periodicity, and long-term periodicity. The residual unit module consists of repeated residual units. In each residual unit, the convolution process is repeated twice after the ReLU activation function, where the first convolution is to learn long-distance spatial patterns by non-adjacent filter coefficients. This is called as the Separated Channel structure, which is capable of modeling patterns between non-adjacent grids. The second convolution is a normal convolution for modeling patterns between adjacent grids. This results in the spatially predicted crowd flow pattern through the ReLU activation function, batch normalization^22^, Dropout^23^, CNN and tanh activation functions. We do not include the detailed POI distribution modeling component in our model. While this external factor can improve prediction accuracy for a specific area, it also makes it difficult to apply the model to areas without this information, thereby limiting its versatility. For instance, the Gowalla data used in this study does not contain detailed POI information. Moreover, in real-world scenarios, collecting detailed POI data can be time-consuming and expensive, and in some cases, impossible. In contrast, data from location-based services can generally be obtained more easily. For the aforementioned reasons, we propose that a model devoid of detailed POI information is more pragmatic from the perspective of a real-world business application.

The key contribution of this study lies in our recognition of the hierarchical structural characteristics of the data, leading us to propose a model structure that can maximize its utilization. This is achieved through the addition of 1) a global data generation module and 2) a sub-module 2 for integrating global data with local data. The internal structure of sub-module 2 mirrors that of sub-module 1, which is derived from the baseline model. However, we propose an optimal architecture for their combination. Experimental results indicate that the optimal point for merging the outputs of global and local data modeling is located between the output of the CNN module and the input of the Residual unit module. Consequently, the proposed model, as depicted in Fig. 1, demonstrates superior performance.

## Experiment

### Data

In this study, we use data from location-based social network services, Foursquare^24^ and Gowalla^25^, which are suitable to test the proposed approach because they can be divided into subgroups by the types of visits. Furthermore, it is possible to conduct experiments by changing the scope of the target area because Gowalla data are collected all over the world.

**Table 1:** Dataset statistics.

Dataset	Tokyo (Foursquare)	New York (Foursquare)	Global (Gowalla)
Users	1939	824	319,063
Venues	61,858	38,336	2,844,076
Check-ins	573,703	227,428	36,001,959
Categories	10	10	7

As can be seen from the Table 1, Foursquare data are collected in Tokyo and New York for about 10 months from April 12, 2012 to February 16, 2013, and include check-in location and time, and ID and type of check-in location. The type of place is divided into a total of 10 sub-types: Arts & Entertainment, College & University, Event, Food, Nightlife Spot, Outdoors & Recreation, Professional & Other Places, Residence, Shop & Service, Travel & Transport. 573,703 check-ins in Tokyo and 227,428 check-ins in New York are included. Gowalla data are collected for about 7 months from November 2010 to May 2011 and consists of 36,001,959 check-ins by 319,063 users in 2,844,076 locations around the world. They can be divided into 7 sub-types: Community, Entertainment, Food, Nightlife, Outdoors, Shopping, and Travel.

### Preparation

To convert check-in data into grid structure-based crowd density data, the entire range of latitude and longitude of data set is divided into a number of grid, and the number of check-ins in each grid is calculated. In Foursquare Tokyo and New York, the entire coverage of the city is divided into 168 grids, resulting that each grid covers about 3 square kilometers which is approximately similar with the coverage of a government administrative region. The Gowalla data come from all around the world which is a wide range of area, and are further tested by dividing into two clusters, Gowalla America and Gowalla Asia/Europe/Africa, etc. K-means clustering is used for dividing entire data set into two clusters. Two types of global data are consisted, one includes all categories, while the other excludes low correlated categories. The prediction performance when using those two global data is compared.

### Training

The ratio of the training data to the test data is 3:1. Mean Square Error is used as the loss function, and Root Mean Square Error is adopted as the accuracy evaluation metric. In this experiment, the most commonly used RMSE index is used because it focuses on comparing the prediction performance between models. We update the weights through the Adam optimization algorithm. The dropout rate is 0.1, and the number of residual units is set to 10. The learning rate is $10^{-4}$, the number of epochs is 800, and the batch size is 32.

### Inference

The final performance is compared by using the test data which is not used for training. The final RMSE is calculated by averaging the RMSEs except for the maximum/minimum RMSE and by repeating the experiment 10 times using the model trained with the training data. Root mean square error is used as performance metric, and RMSE difference of less than 5% is judged to be equivalent.

This study conducted performance comparison research with a baseline model that does not include the proposed techniques, in order to validate the effects of proposed model. Additionally, as part of the ablation experiment, comparative experiments were conducted considering and not considering correlation when generating global data. These results are described in the following section. Also, the qualitative analysis of the experimental results is described in the following section.

## Result

In the RMSE comparison table, ’Model (A)’ uses global data composed of all other categories, while ’Model (B)’ uses global data consisting only of highly correlated categories with the target category, which is marked in bold in the correlation table.

**Table 2:** Foursquare Tokyo—Correlation between categories.

Cat.	#1	#2	#3	#4	#5	#6	#7	#8	#9	#10
#1	1.000	**0.335**	0.165	**0.476**	0.150	**0.311**	**0.330**	0.131	**0.462**	**0.479**
#2	0.335	1.000	0.134	0.272	0.080	0.168	0.208	0.073	0.285	0.303
#3	0.165	0.134	1.000	0.241	0.093	0.124	0.112	− 0.008	0.121	0.242
#4	**0.476**	0.272	0.241	1.000	0.197	**0.428**	**0.445**	0.153	**0.596**	**0.651**
#5	0.150	0.080	0.093	0.197	1.000	0.128	0.121	0.064	0.171	0.201
#6	0.311	0.168	0.124	0.428	0.128	1.000	0.381	0.113	0.415	0.413
#7	**0.330**	0.208	0.112	**0.445**	0.121	**0.381**	1.000	0.099	**0.430**	**0.442**
#8	0.131	0.073	-0.008	0.153	0.064	0.113	0.099	1.000	0.153	0.161
#9	**0.462**	0.285	0.121	**0.596**	0.171	**0.415**	**0.430**	0.153	1.000	**0.605**
#10	**0.479**	0.303	0.242	**0.651**	0.201	**0.413**	**0.442**	0.161	**0.605**	1.000

**Table 3:** Foursquare Tokyo—RMSE comparison.

Cat.	STResNet	DeepSTN+	Model(A)	RMSE $\downarrow$	Model(B)	RMSE $\downarrow$
#1	0.0479	0.0406	0.0384	5.4%	**0.0379**	**6.7%**
#2	0.0289	0.0274	0.0269	–	0.0269	–
#3	0.0146	0.0146	0.0146	–	0.0146	–
#4	0.0581	0.0409	0.0384	6.1%	**0.038**	**7.1**%
#5	0.0409	0.0408	0.0409	–	0.0409	–
#6	0.0107	0.01	0.01	–	0.01	–
#7	0.0412	0.036	**0.034**	**5.6%**	**0.034**	**5.6%**
#8	0.042	0.042	0.042	–	0.042	–
#9	0.0357	0.0285	0.0255	10.5%	**0.0254**	**10.9%**
#10	0.0667	0.0449	**0.043**	**6.9%**	**0.043**	**6.9%**

### Foursquare Tokyo

Table 2 presents the correlation of data across categories, which significantly impacts the performance of the proposed model. Table 3 illustrates the prediction accuracy of both the baseline and proposed models. The performance improvement of the proposed method over other methods can be attributed to its unique approach. To predict one of the data subcategories, unlike existing models that typically rely solely on the target category data, our method employs a combination of both aggregated (global) and segmented (local) data.

From Table 2, it can be observed that the overall correlation ranges from − 0.008 to 0.651. The correlation between categories, on the whole, is not particularly strong, with the highest correlation being 0.651. This is also reflected in the RMSE comparison results in Table 3, which demonstrate that the performance improvement is not substantial when using global data. Specifically, when the correlation between categories is higher, the prediction performance improves more significantly by incorporating global data.

Category 1 (Arts & Entertainment) exhibits a correlation of $0.131-0.479$ with other categories, and it is observed that 6 categories have correlations higher than the correlation threshold of N(0.31), which is relatively high in this case. This correlation pattern results in a 5.4% reduction in error when all categories are included as global data. Furthermore, when experiments are conducted using global data but categories 3, 5, and 8 are excluded, which have relatively low correlation with the target category, an additional 1.3% reduction in error is observed in Table 3. It can be seen that category 4 (Food) and category 9 (Shop & Service) also exhibit a similar trend.

**Figure 2:**
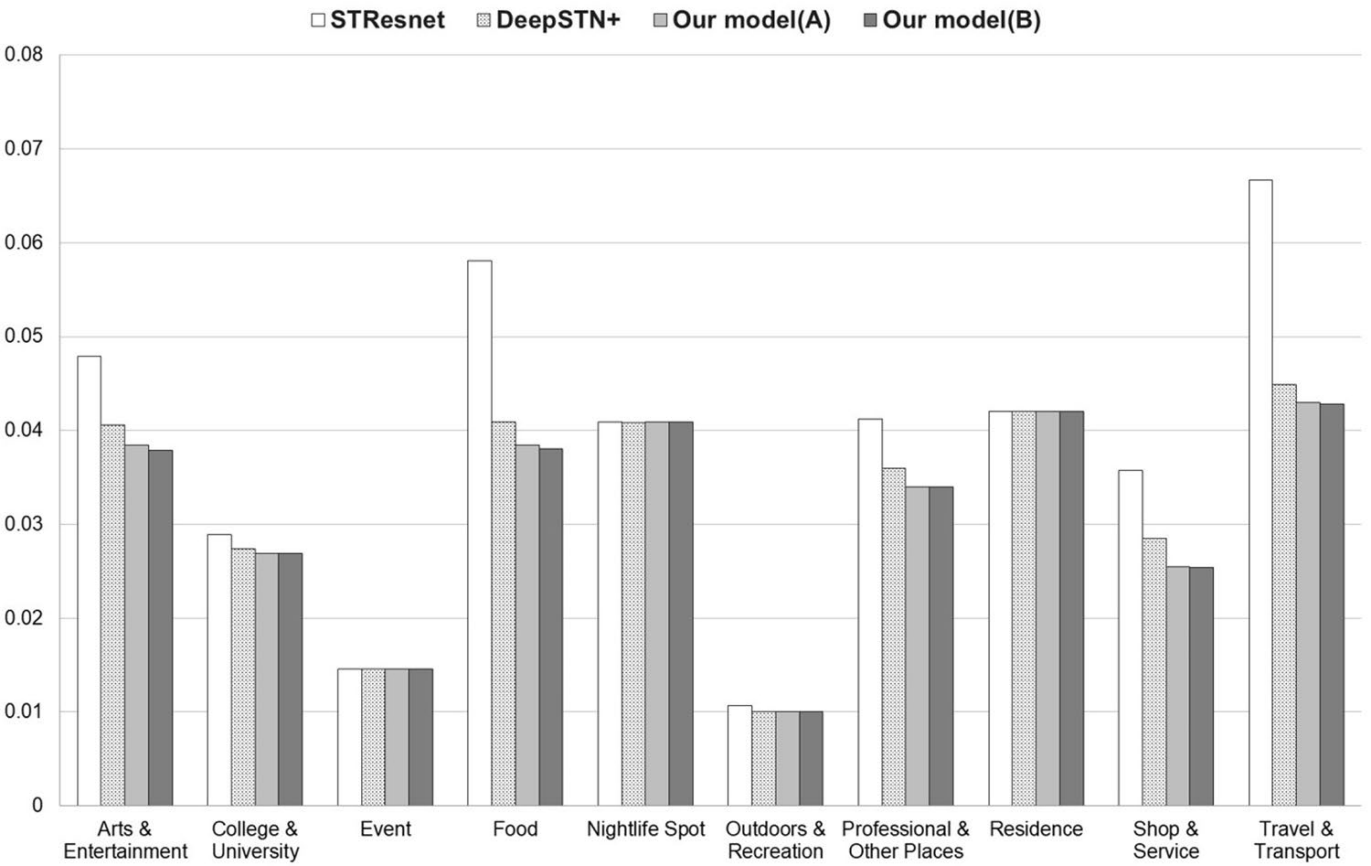
Foursquare Tokyo RMSE comparison.

Regarding Category 7 (Professional & Other Places) and Category 10 (Travel & Transport), it is observed that the prediction error is reduced when configuring global data with all categories, but no additional improvement is observed when global data is configured based on correlation. This requires further analysis.

In the case of categories 2 (College & University), 3 (Event), 5 (Nightlife Spot), 6 (Outdoors & Recreation), and 8 (Outdoors & Recreation), it appears that there is no performance improvement when using global data because the correlation between these categories and other categories is lower than the threshold value of N.

Figure 2 shows that the proposed model provides better performance than base models in Foursquare Tokyo dataset.

### Foursquare New York

Table 4 shows that the correlation between categories ranges from -0.008 to 0.655. Overall, the correlation between categories is not particularly strong, with the maximum correlation being 0.655, which is also reflected in the RMSE performance results in Table 5. The performance improvement ranges from 5.2 to 6.96%, as shown in Table 5, which is less significant compared to the performance gain observed in the Gowalla data, as discussed later. In addition, the performance improvement tends to be proportional to the correlation between categories. Category 1 (Arts & Entertainment) shows a correlation of 0.086 to 0.589, and 5 categories have a significant correlation with Category 1 higher than the threshold of N(0.31).

This results in a 6.1% reduction in error when all categories are included as global data. Furthermore, when configuring the global data by excluding categories 2, 3, 5, and 8, which have relatively low correlation, an additional 0.7% reduction in error is achieved.

Categories 6 (Outdoors & Recreation), 7 (Professional), and 9 (Shop & Service) also exhibit similar trends.

Regarding Category 4 (Food), while including all categories in global data results in a 6.4% reduction in errors, no further improvement is observed when global data is configured with consideration of correlation. Similarly, for Category 10 (Travel & Transport), no performance gain is observed when using global data, and this requires further investigation.

**Table 4:** Foursquare New York—Correlation between categories.

Cat.	#1	#2	#3	#4	#5	#6	#7	#8	#9	#10
#1	1.000	0.282	0.202	**0.589**	0.195	**0.444**	**0.400**	0.086	**0.474**	**0.427**
#2	0.282	1.000	0.105	0.283	0.078	0.236	0.242	0.052	0.254	0.212
#3	0.202	0.105	1.000	0.245	0.068	0.174	0.180	− 0.008	0.199	0.159
#4	**0.589**	0.283	0.245	1.000	0.219	**0.601**	**0.577**	0.124	**0.655**	**0.567**
#5	0.195	0.078	0.068	0.219	1.000	0.188	0.151	0.016	0.191	0.201
#6	**0.444**	0.236	0.174	**0.601**	0.188	1.000	**0.494**	0.102	**0.524**	**0.495**
#7	**0.400**	0.242	0.180	**0.577**	0.151	**0.494**	1.000	0.105	**0.514**	**0.454**
#8	0.086	0.052	-0.008	0.124	0.016	0.102	0.105	1.000	0.118	0.087
#9	**0.474**	0.254	0.199	**0.655**	0.191	**0.524**	**0.514**	0.118	1.000	**0.507**
#10	**0.427**	0.212	0.159	**0.567**	0.201	**0.495**	**0.454**	0.087	**0.507**	1.000

**Table 5:** Foursquare New York—RMSE comparison.

Cat.	STResNet	DeepSTN+	Model(A)	RMSE $\downarrow$	Model(B)	RMSE $\downarrow$
#1	0.0163	0.0147	0.0138	6.1%	**0.0136**	**6.8%**
#2	0.029	0.0283	0.0283	–	0.0283	–
#3	0.0152	0.0152	0.0152	–	0.0152	–
#4	0.0301	0.0205	**0.0192**	**6.4%**	**0.0192**	**6.4%**
#5	0.0063	0.0063	0.0063	–	0.0063	–
#6	0.0178	0.0158	0.0149	5.7%	**0.0147**	**6.96%**
#7	0.0229	0.0192	**0.0182**	**5.3%**	**0.018**	**6.25%**
#8	0.0253	0.0253	0.0252	–	0.0252	–
#9	0.0267	0.0191	0.0181	5.2%	**0.0179**	**6.3%**
#10	0.0209	0.0166	**0.0164**	–	**0.0165**	–

### Gowalla global

Looking at the correlation analysis results between categories in Table 6, it can be seen that the overall correlations range from a minimum of 0.775 to a maximum of 0.906. Considering that the correlation between categories in Foursquare data is up to 0.65, we can conclude that the correlations between categories in Gowalla are generally stronger than those in Foursquare. Looking at the experimental results in Table 7, the error reduction for Gowalla ranges from 6.3 to 36.1%, which is significantly higher than that of Foursquare, which ranges from $\sim 10.9\%$. Once again, we see that the performance improvement when using global data tends to be proportional to the correlation between categories.

Additionally, the differences in correlation between categories in Gowalla are smaller than those in Foursquare. Thus, we can expect that the performance improvement when global data is configured considering correlation would be smaller in Gowalla than in Foursquare.

When composing global data by considering correlation in Foursquare, there are 5 categories that show performance improvement. However, in Gowalla, only 2 categories show additional performance improvements. We can infer that as the difference in correlation between categories increases, the performance tends to be better when composing global data considering correlation.

In the case of category 3 (Food), the correlation with other categories ranges from 0.775 to 0.906, and the difference between the maximum and minimum values of correlation is 0.131, which is relatively high compared to other categories in Gowalla global. It can be expected that there will be additional performance improvement when global data is configured considering correlation. As expected, the experiment results show that the prediction error is 7.55% lower when global data is configured considering correlation. Category 7 (Travel) also shows a similar trend.

**Table 6:** Gowalla Global—Correlation between categories.

Category	#1	#2	#3	#4	#5	#6	#7
#1_Community	1.000	0.838	**0.844**	0.818	**0.856**	**0.871**	**0.852**
#2_Entertainment	**0.838**	1.000	0.816	**0.818**	**0.854**	**0.865**	0.815
#3_Food	**0.844**	**0.816**	1.000	0.802	**0.809**	**0.906**	0.775
#4_Nightlife	**0.818**	**0.818**	0.802	1.000	**0.806**	**0.840**	0.778
#5_Outdoor	0.856	0.854	0.809	0.806	1.000	0.845	0.815
#6_Shopping	0.871	0.865	0.906	0.840	0.845	1.000	0.824
#7_Travel	**0.852**	**0.815**	0.775	0.778	**0.815**	**0.824**	1.000

**Table 7:** Gowalla Global—RMSE comparison.

Cat.	STResNet	DeepSTN+	Model(A)	RMSE $\downarrow$	Model(B)	RMSE $\downarrow$
#1	0.0293	0.0135	**0.012**	**11.11%**	**0.012**	**11.11%**
#2	0.0091	0.008	**0.0075**	**6.3%**	**0.0075**	**6.3%**
#3	0.0173	0.0119	0.0107	10.1%	**0.0098**	**17.65%**
#4	0.0065	0.0054	**0.005**	**7.4%**	**0.005**	**7.4%**
#5	0.0133	0.0097	0.0095	–	0.0095	–
#6	0.0148	0.0077	0.0074	–	0.0074	–
#7	0.0101	0.0097	0.0066	32%	**0.0062**	**36.1%**

**Figure 3:**
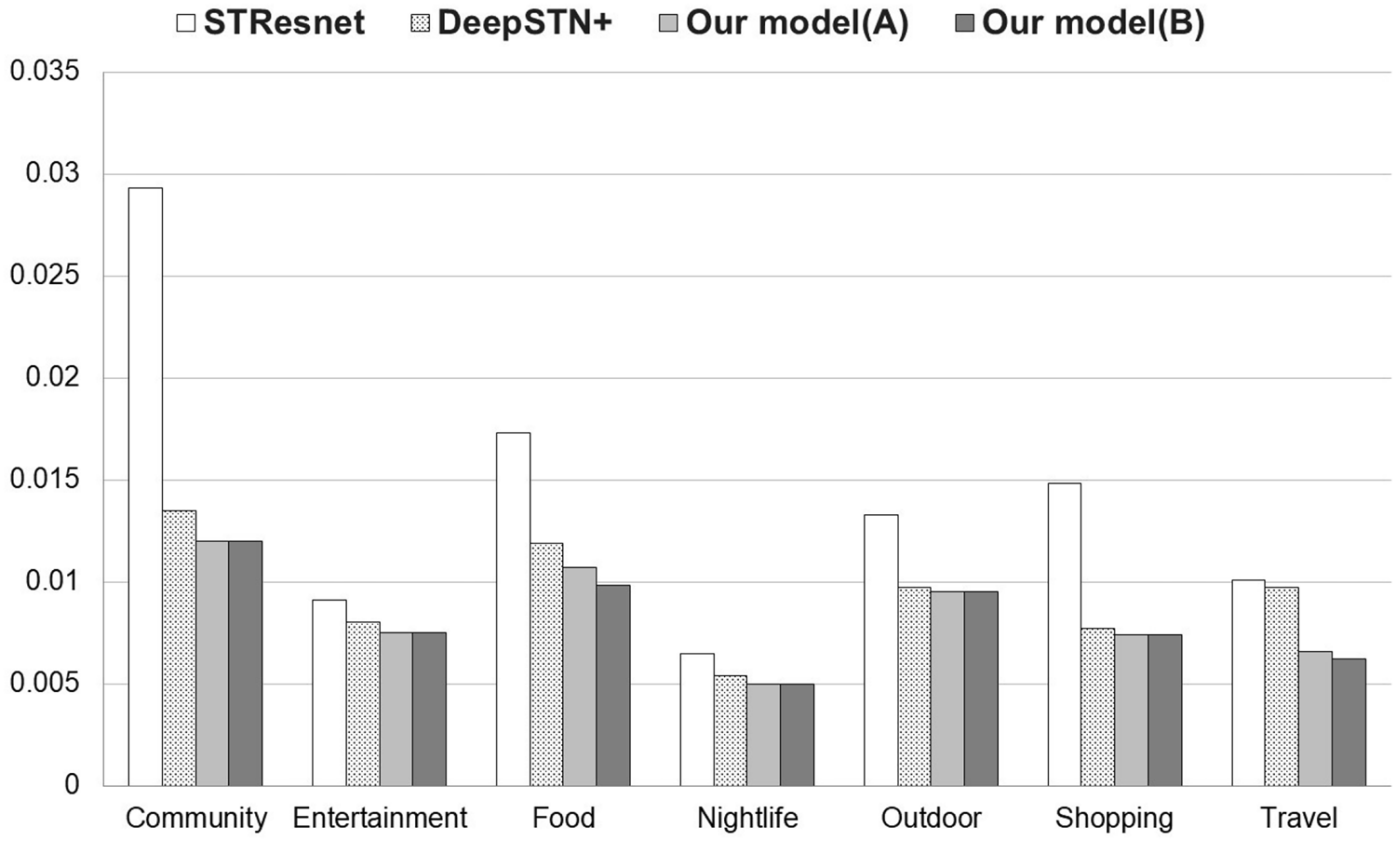
Gowalla global RMSE comparison.

In categories 1 (Community), 2 (Entertainment), and 4 (Nightlife), the correlation between categories is high, and the difference in correlation between categories is also relatively small. This may explain why there is no additional performance improvement observed when configuring global data with consideration of correlation in these categories.

Figure 3 shows that the proposed model provides better performance than base models in Gowalla Global dataset.

### Gowalla America

Looking at the correlation analysis results between categories in Table 8, it can be seen that the overall correlations range from 0.797 to 0.9. From the experimental results in Table 9, the performance improvement is from 12.5 to 43.5%, which is higher than the performance improvement of Gowalla global. The reason for this, regardless of no significant difference in the correlations, would be inferred from the amount of data. In the case of Gowalla America, the number of samples is smaller because only samples from the Americas region are selected from Gowalla global. It can be seen that the smaller the number of samples, the better the performance improvement of using global data including all the other categories.

**Table 8:** Gowalla America—Correlation between categories.

Category	#1	#2	#3	#4	#5	#6	#7
#1_Community	1.000	**0.900**	0.838	0.833	**0.875**	**0.881**	**0.856**
#2_Entertainment	**0.900**	1.000	0.803	**0.846**	**0.885**	**0.856**	0.844
#3_Food	**0.838**	0.803	1.000	**0.810**	0.797	**0.869**	**0.829**
#4_Nightlife	**0.833**	**0.846**	0.810	1.000	**0.811**	**0.839**	0.802
#5_Outdoor	**0.875**	**0.885**	0.797	0.811	1.000	**0.844**	**0.837**
#6_Shopping	**0.881**	**0.856**	**0.869**	**0.839**	**0.844**	1.000	**0.853**
#7_Travel	**0.856**	**0.844**	0.829	0.802	**0.837**	**0.853**	1.000

**Table 9:** Gowalla America—RMSE comparison.

Cat.	STResNet	DeepSTN+	Model(A)	RMSE $\downarrow$	Model(B)	RMSE $\downarrow$
#1	0.0245	0.0177	0.011	37.85%	**0.01**	**43.50%**
#2	0.0093	0.0087	0.0071	18.4%	**0.0064**	**26.4%**
#3	0.0134	0.0123	0.01	18.70%	**0.009**	**26.83%**
#4	0.0091	0.0088	0.0062	29.5%	**0.006**	**31.8%**
#5	0.0235	0.0159	0.013	18.24%	**0.0129**	**18.24%**
#6	0.0132	0.0128	0.0104	18.75%	**0.010**	**18.75%**
#7	0.0106	0.0096	**0.0084**	**12.5%**	**0.0084**	**12.5%**

In Gowalla America data, the correlations between the target measurement category and other categories are high overall, and the differences in the correlation between categories are small. Therefore, when global data is composed of all categories, the performance improves significantly, whereas when composing global data considering correlation, additional performance improvement is not significant.

**Figure 4:**
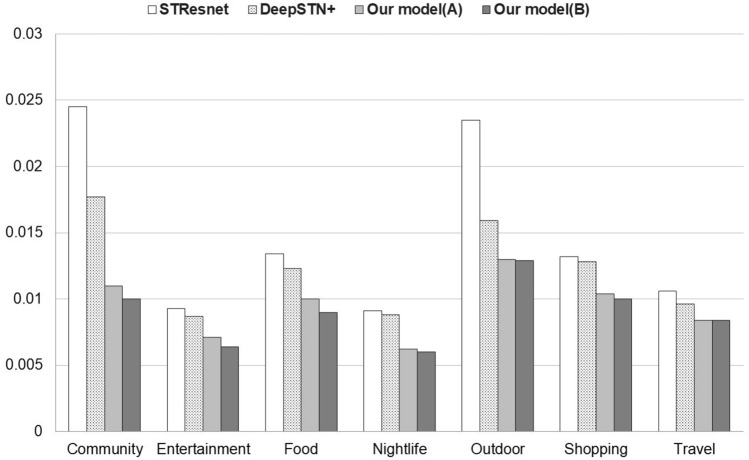
Gowalla America RMSE comparison.

This can be confirmed from the experimental results in Table 9. When global data is composed of all categories, the average error reduction is 22%, which is 8.6% higher than that of Gowalla Global (13.4%). When the global data is configured in consideration of correlation, there is a slight improvement, but the RMSEs themselves are not significantly different.

Figure 4 shows that the proposed model provides better performance than base models in Gowalla America dataset.

### Gowalla Asia/Europe/Africa, etc

Looking at the correlation analysis results between categories in Table 10, it can be seen that the overall correlations range from 0.734–0.921. According to the experimental results in Table 11, the RMSE reduction of Gowalla Asia/Europe/Africa is from 9.71 to 48.65%, which is similar to the performance improvement level of Gowalla America.

**Table 10:** Gowalla Asia/Europe/Africa, etc.—Correlation between categories.

Category	#1	#2	#3	#4	#5	#6	#7
#1_Community	1.000	0.791	0.857	0.782	**0.801**	**0.866**	0.756
#2_Entertainment	**0.791**	1.000	**0.807**	0.740	**0.795**	**0.799**	0.745
#3_Food	**0.857**	0.807	1.000	**0.822**	**0.822**	**0.921**	0.778
#4_Nightlife	**0.782**	**0.740**	**0.822**	1.000	0.739	**0.817**	0.734
#5_Outdoor	**0.801**	**0.795**	**0.822**	0.739	1.000	**0.802**	0.755
#6_Shopping	**0.866**	0.799	**0.921**	**0.921**	**0.802**	1.000	0.770
#7_Travel	**0.756**	0.745	**0.778**	0.734	**0.755**	**0.770**	1.000

**Table 11:** Gowalla Asia/Europe/Africa, etc—RMSE comparison.

Cat.	STResNet	DeepSTN+	Model(A)	RMSE $\downarrow$	Model(B)	RMSE $\downarrow$
#1	0.0095	0.0078	0.0078	–	0.0075	–
#2	0.0105	0.0099	**0.0078**	**21.21%**	**0.0078**	**21.21%**
#3	0.0132	0.0103	0.0093	9.71%	**0.0079**	**23.30%**
#4	0.0069	0.0053	0.0051	–	**0.0045**	**15.09%**
#5	0.0084	0.0074	0.004	45.95%	**0.0038**	**48.65%**
#6	0.0096	0.008	0.0072	10.00%	**0.0068**	**15.00%**
#7	0.0076	0.0067	0.0039	41.79%	**0.0036**	**46.27%**

**Figure 5:**
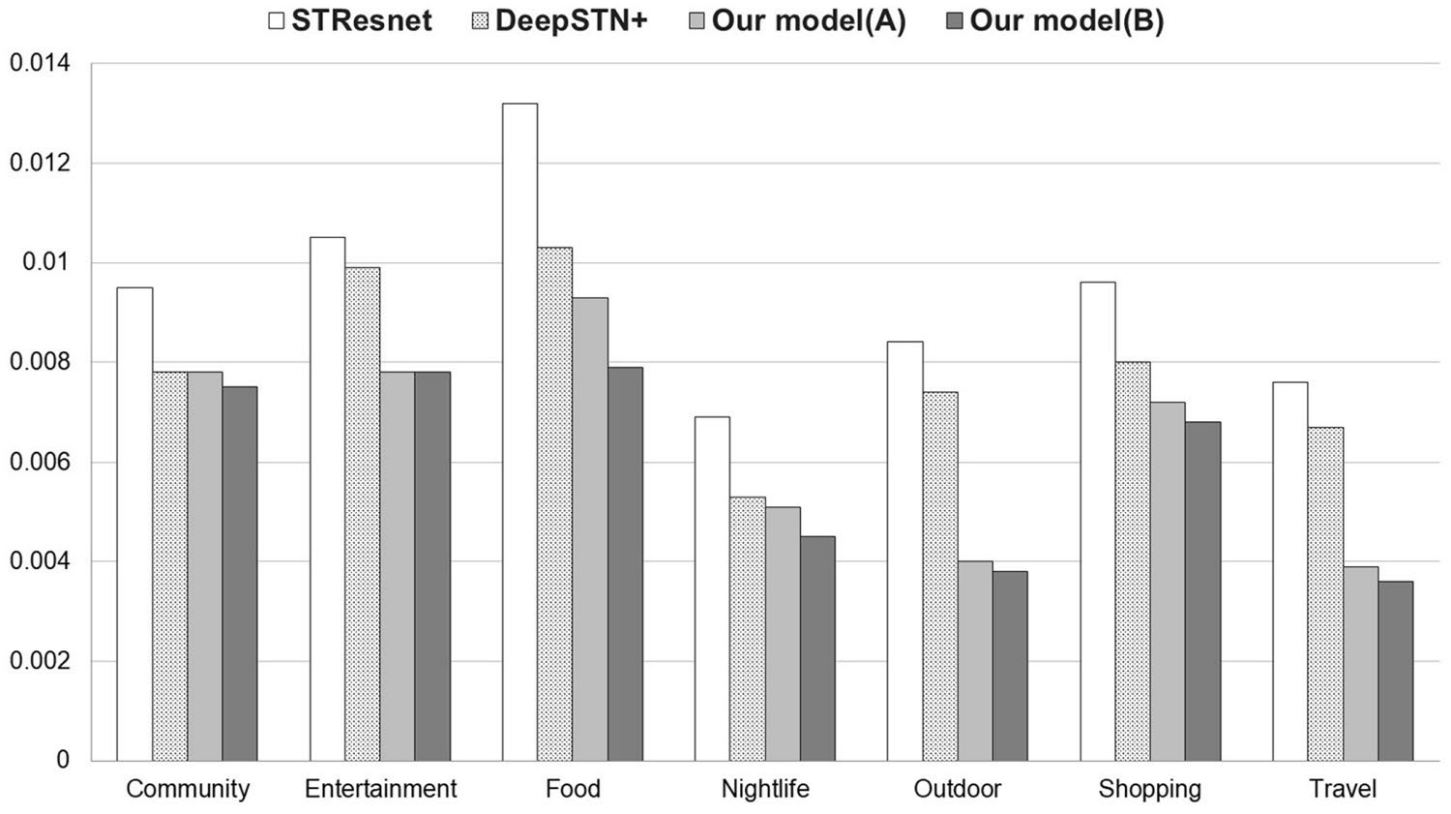
Gowalla Asia/Europe/Africa, etc RMSE comparison.

Figure 5 shows that the proposed model provides better performance than base models in Asia/Europe/Africa dataset.

## Conclusion

In this study, we propose a deep learning model that takes into account the global-local structure of crowd flow data. The model utilizes both overall (global) and subdivided (local) crowd flow data simultaneously, with the goal of increasing the prediction accuracy for each sub-divided subgroup. Furthermore, with respect to global data, we propose a method that takes into account not only all categories but also the correlation between the prediction target category and each other category. Specifically, we exclude categories that have a low correlation with the target category from global data. The experiment result shows that the proposed model improves the prediction accuracy of each sub-divided subgroup by 5.2% (Table 5. Cat #9)—45.95% (Table 11. Cat #5), depending on the data set. This result comes from the comparison with the related works under the same condition that use target category data to predict each subgroup. In addition, when we advance the global data composition by considering the correlation between subgroups, which excludes low correlated subgroups from global data, the prediction accuracy is even more improved by 5.6% −  48.65%. Overall, if the correlation between the prediction category and other categories is high, the approach of combining local and global data shows high performance improvement. In principle, the entire crowd flow(global) data should be correlated with each category to some extent, which means that the proposed approach can be generalized to all kinds of crowd flow data. Furthermore, the overall performance is enhanced when the global data is configured by considering correlation, which is excluding categories with relatively low correlation. The approach of excluding categories with low correlation to the target category from the global data tends to improve prediction performance more as the correlation between the target category and other categories decreases. This study can be utilized in various purpose such as tailored marketing, new drug development and so on.

## Data Availability

Our code for the experiments is available at https://github.com/ilyhs79/Glocal_STN.
