# Author Correction: A study on deep learning model based on global–local structure for crowd flow prediction

**DOI:** 10.1038/s41598-024-66605-w

**Published:** 2024-07-08

**Authors:** HeounMo Go, SangHyun Park

**Affiliations:** Department of Computer Science, Yonsei University, Yonsei-ro 50, Seodaemun-gu, Seoul, 03722 Republic of Korea

Correction to: *Scientific Reports* 10.1038/s41598-024-63310-6, published online 01 June 2024

In the original version of this Article HeounMo Go was incorrectly affiliated with ‘AI Service Division, SK Telecom, Eulji‑ro 65, Jung‑gu, Seoul 04539, Republic of Korea’. The correct affiliation is listed below.

Department of Computer Science, Yonsei University, Yonsei‑ro 50, Seodaemun‑gu, Seoul 03722, Republic of Korea.

The original Article has been corrected.

